# Microbiological Research on Fermented Dairy Products

**DOI:** 10.3390/foods11142109

**Published:** 2022-07-15

**Authors:** Luyao Xiao, Wei Li

**Affiliations:** College of Food Science and Technology, Nanjing Agricultural University, Nanjing 210095, China; 2021208015@stu.njau.edu.cn

Fermented dairy products are widely consumed worldwide due to the nutritional and health benefits. Microorganisms play an extremely important role in fermented dairy products, which are essential for obtaining final products with well-defined organoleptic and physico-chemical properties. Microbial ecology of dairy products is composed of complex microbiota, including bacteria, yeasts and filamentous fungi. Recently, particular attention has been given to the extracellular biopolymers called exopolysaccharides (EPS) isolated from lactic acid bacteria (LAB) owing to their prebiotic properties and applicability in the food industry. Furthermore, gastro-intestinal digestion of dairy proteins is an important step to supply the organism of essential and non-essential amino acids, and thus peptidomics approach has proven to be a suitable method to monitor hydrolysis during digestion of diary proteins.

This Special Issue belongs to the section “Food Microbiology” and focuses on the microbiota diversity, carbohydrate metabolism, peptidomic pattern and quality improvement of fermented dairy products. This Special Issue includes six research papers related to microbiological research on fermented dairy products, all of which are valuable contributions to this issue made by distinguished experts and researchers in this area.

Wang et al. (2021) [1] analyzed bacterial diversity of traditional Tibetan kefir grains. Kefir grains are made up of complex microbiota including LAB, yeast and acetic acid bacteria (AAB). *Lactobacillus kefiranofaciens*, *L. kefiri* and *Kluyveromyces marxianus* were identified as the main dominant strains in the kefir grains. The authors investigated the chemical structure of EPS and biofilm formation to clarify the internal relationship between biofilm and the formation process of kefir grains.

The dominant microbiota of four Traditional Agri-food Products (TAP) cheeses as well as their peptidase activities were studied by Celano et al. (2022) [2]. The authors concluded that *L. helveticus* dominated both Caprino and Vaccino cheeses, while *Staphylococcus equorum* and *Streptococcus thermophilus* dominated Cacioricotta and Pecorino cheeses, respectively. Potential relationship between the microbiological and their biochemical characteristics were predicted for valorizing and safeguarding these TAP cheeses.

An EPS was extracted from soybean whey fermented by *Lactiplantibacillus plantarum* 70810. The preliminary structural characteristics and antioxidant activity of the EPS, that could be used as a promising ingredient for health-beneficial function foods, were examined by Tian et al. (2021) [3]. Soybean whey is a by-product of the soybean production and could be transformed into bioactive ingredients by microbial fermentation. The authors suggested that EPS from soybean whey fermented by *L. plantarum* 70810 exhibited great scavenging bioactivities against the DPPH radical and superoxide anion radical.

Ye et al. (2021) [4] reported the peptidomics of milk fermented by *Lactobacillus delbrueckii* both before and after the simulated gastrointestinal digestion. The peptides derived from β-casein were abundant in the samples of *L. delbreuckii* ssp. *bulgaricus*, while the peptides originating from αs1-casein and αs2-casein were more likely to dominate in those of *L. delbrueckeii* ssp. *lactis*. The research provided a reference approach for evaluating the peptide profile of the *Lactobacillus delbrueckii* during fermentation and digestion.

The physicochemical, rheological and microstructural properties of non-fat yogurt fortified with whey protein isolate was examined by Hashim et al. (2021) [5] to explore the potential of whey protein isolate (WPI) in improving the yogurt quality compared with skim milk powder (SMP). The authors concluded that the incorporation of WPI improved the water holding capacity, rheological properties, compared sensory and textural characteristics, and could be further exploited for producing low- and non-fat fermented dairy food products.

A unique gene (the glycosyltransferase family 8) was identified to differentiate *Lacticaseibacillus zeae* from other *Lacticaseibacillus* species by pan-genome analysis, and a real-time PCR method was developed and applied to accurately detect the unique gene by Kim et al. (2021) [6]. The traditional method for differentiation of lactic acid bacteria is based on the highly conservative 16S rRNA gene. The development of this technology could facilitate the identification of specific strains among its closely related species.

The manuscripts in this Special Issue deal with the undeniable importance of microbiota for the fermented fairy products. Further studies are required to obtain a better understanding of how fermented dairy products provide health benefits, and more attention should be paid to the mechanisms by which organisms, cellular fractions or the byproducts are responsible for each benefit.
